# A dynamic model of gene expression in monocytes reveals differences in immediate/early response genes between adult and neonatal cells

**DOI:** 10.1186/1476-9255-4-4

**Published:** 2007-02-16

**Authors:** Shelley Lawrence, Yuhong Tang, M Barton Frank, Igor Dozmorov, Kaiyu Jiang, Yanmin Chen, Craig Cadwell, Sean Turner, Michael Centola, James N Jarvis

**Affiliations:** 1 Dept. of Pediatrics, Neonatal Section, University of Oklahoma College of Medicine, Oklahoma City, OK, USA; 2 Arthritis & Immunology Program Oklahoma Medical Research Foundation, Oklahoma City, 73104, USA

## Abstract

Neonatal monocytes display immaturity of numerous functions compared with adult cells. Gene expression arrays provide a promising tool for elucidating mechanisms underlying neonatal immune function. We used a well-established microarray to analyze differences between LPS-stimulated human cord blood and adult monocytes to create dynamic models for interactions to elucidate observed deficiencies in neonatal immune responses.

We identified 168 genes that were differentially expressed between adult and cord monocytes after 45 min incubation with LPS. Of these genes, 95% (159 of 167) were over-expressed in adult relative to cord monocytes. Differentially expressed genes could be sorted into nine groups according to their kinetics of activation. Functional modelling suggested differences between adult and cord blood in the regulation of apoptosis, a finding confirmed using annexin binding assays. We conclude that kinetic studies of gene expression reveal potentially important differences in gene expression dynamics that may provide insight into neonatal innate immunity.

## Background

The defects in neonatal adaptive immunity are relatively easy to understand *a priori*. Although there are complexities to be considered [1,2], experimental evidence demonstrates that newborns, lacking prior antigen exposure, must develop immunologic memory based on postnatal experience with phogens and environmental immunogens [3-5].

It is less clear why there should be defects in newborns' innate immunity, although these defects are well documented. For example, newborns have long been known to exhibit defects in phagocytosis [6], chemotaxis [7,8], and adherence [9], the latter possibly due to aberrant regulation of critical cell-surface proteins that mediate leukocyte-endothelial interactions [10]. Newborn monocytes also exhibit diminished secretion of numerous cytokines under both stimulated and basal conditions [11-13].

Elucidating the causes of these defects is a crucial question in neonatal medicine, since infection remains a major cause of morbidity and mortality in the newborn period. However, unravelling the complex events in monocyte and/or neutrophil activation, from ligand binding to activation of effector responses, is clearly a daunting challenge. Any one of numerous pathways from the earliest cell signalling events to protein synthesis or secretion could be relevant, and focusing on any one may overlook critical aspects of cellular regulation. In this context, genomic and/or proteomic approaches may offer some important advantages, at least in the initial phases of investigation, by allowing investigators to survey the panoply of biological processes that may be relevant to identifying critical biological distinctions.

Recently published work has documented differences in gene expression between adult and cord blood monocytes [14], although these studies did not elucidate the fundamental, functional differences between cord blood and adult cells. The studies we report here demonstrate how computational analyses, applied to microarray data, can elucidate critical biological functions when analysis extends beyond the identification of differentially-expressed genes.

## Methods

### Cells and cellular stimulation

Monocytes were purified from cord blood of healthy, term infants and from the peripheral blood of healthy adults by positive selection using anti-CD-14 mAb-coated magnetic beads (Miltenyi Biotec, Auburn, CA, USA) according to the manufacturer's instructions. Informed consent was obtained from adult volunteers; collection of cord blood was ruled exempt from consent after review by the Oklahoma Health Sciences Center IRB. In brief, blood was collected into sterile tubes containing sodium citrate as an anticoagulant (Becton Dickinson, Franklin Lakes, NJ). Peripheral blood mononuclear cells (PBMC) were prepared from the anti-coagulated blood using gradient separation on Histopaque-1077 performed directly in the blood collection tubes. Cells were washed three times in Ca^2+ ^and Mg^2+^-free Hanks's balanced salt solution. PBMC were incubated for 20 min at 4°C with CD14 microbeads at 20 *μ*l/1 × 10^7 ^cells. The cells were washed once, re-suspended in 500 *μ*l Ca^2+ ^and Mg^2+^-free PBS containing 5% FBS/1 × 10^8 ^cells. The suspension was then applied to a MACs column. After unlabeled cells passed through, the column was washed with 3 × 500 μl Ca^2+ ^and Mg^2+^-free PBS. The column was removed from the separator and was put on a new collection tube. One ml of Ca^2+ ^and Mg^2+^-free PBS was then added onto the column, which was immediately flushed by firmly applying the plunger supplied with the column.

Purified monocytes were incubated with LPS from *Escherichia coli *0111:4B (Sigma, St. Louis, MO) at 10 ng/ml for 45 min and 2-hours in RPMI 1640 with 10% fetal bovine serum or studied in the absence of stimulation ("zero time"). It should be noted that this product is not "pure," and stimulates both TLR-4 and TRL-2 signaling pathways [15]. A smaller number of replicates (n = 5) was analyzed after 24 hr incubation. After the relevant time points, monocytes were lysed with TriZol (Invitrogen, Carlsbad, CA, USA) and RNA was isolated as recommended by the manufacturer. Cells from eight different term neonates and eight different healthy adults were used for these studies.

### Gene microarrays

The microarrays used in these experiments were developed at the Oklahoma Medical Research Foundation Microarray Research Facility and contained probes for 21,329 human genes. Slides were produced using commercially available libraries of 70 nucleotide long DNA molecules whose length and sequence specificity were optimized to reduce the cross-hybridization problems encountered with cDNA-based microarrays (Qiagen-Operon). The oligonucleotides were derived from the UniGene and RefSeq databases. The RefSeq database is an effort by the NCBI to create a true reference database of genomic information for all genes of known function. All 11,000 human genes of known or suspected function were represented on these arrays. In addition, most undefined open reading frames were represented (approximately 10,000 additional genes).

Oligonucleotides were spotted onto Corning^® ^UltraGAPS™ amino-silane coated slides, rehydrated with water vapor, snap dried at 90°C, and then covalently fixed to the surface of the glass using 300 mJ, 254 nm wavelength ultraviolet radiation. Unbound free amines on the glass surface were blocked for 15 min with moderate agitation in a 143 mM solution of succinic anhydride dissolved in 1-methyl-2-pyrolidinone, 20 mM sodium borate, pH 8.0. Slides were rinsed for 2 min in distilled water, immersed for 1 min in 95% ethanol, and dried with a stream of nitrogen gas.

### Labeling, hybridization, and scanning

Fluorescently labeled cDNA was separately synthesized from 2.0 μg of total RNA using an oligo dT_12–18 _primer, PowerScript reverse transcriptase (Clontech, Palo Alto, CA), and Cy3-dUTP (Amersham Biosciences, Piscataway, NJ) for 1 hour at 42°C in a volume of 40 μl. Reactions were quenched with 0.5 M EDTA and the RNA was hydrolyzed by addition of 1 M NaOH for 1 hr at 65°C. The reaction was neutralized with 1 M Tris, pH 8.0, and cDNA was then purified with the Montage PCR_96 _Cleanup Kit (Millipore, Billerica, MA). cDNA was added to ChipHybe™ hybridization buffer (Ventana Medical Systems, Tucson, AZ) containing Cot-1 DNA (0.5 mg/ml final concentration), yeast tRNA (0.2 mg/ml), and poly(dA)_40–60 _(0.4 mg/ml). Hybridization was performed on a Ventana Discovery system for 6 hr at 42°C. Microarrays were washed to a final stringency of 0.1× SSC, and then scanned using a dual-color laser (Agilent Biotechnologies, Palo Alto, CA). Fluorescent intensity was measured by Imagene™ software (BioDiscovery, El Segundo, CA).

### PCR validation of array data

#### Reverse transcription

Three cord blood samples (C1, C2, and C5) and three adult samples (A1, A5, and A6) from the 45 minute time point were used for PCR validation. First strand cDNA was generated from 3.6 μg of total RNA per sample using the OmniScript Reverse Transcriptase and buffer (Qiagen, Valencia, CA), 1 μl of 100 μM oligo dT primer (dT_15_) in a 40 μl volume. Reactions were incubated 60 min at 37° and inactivated at 93° for 5 min. cDNA was diluted 1:100 in water and stored at -20°C.

### Quantitative PCR

Gene-specific primers for 10 genes (*Erbb3, Tmod, Dscr1l1, Sp1, Scya4, Gro2, Cri1, Scya3, Scya3l1*, and *Il-1a*) were designed with a 60°C melting temperature and a length of 19–25 bp for PCR products with a length of 90–140 bp, using Applied Biosystems Inc (ABI, Foster City, CA) Primer Express 1.5 software. PCR was run with 2 μl cDNA template in 15 μl reactions in triplicate on an ABI SDS 7700 using the ABI SYBR Green I Master Mix and gene specific primers at a concentration of 1 μM each. The temperature profile consisted of an initial 95°C step for 10 minutes (for Taq activation), followed by 40 cycles of 95°C for 15 sec, 60°C for 1 min, and then a final melting curve analysis with a ramp from 60°C to 95°C over 20 min. Gene-specific amplification was confirmed by a single peak in the ABI Dissociation Curve software. No template controls were run for each primer pair. Since equal amounts of total RNA were used for cDNA synthesis, Ct values should reflect relative abundance [16]. These values were used to calculate the average group Ct (Cord vs. Adult) and the relative ΔCt was used to calculate fold change between the two groups [17].

### Apoptosis assays

Exposed membrane phospholipids (a marker for early apoptosis) were detected in adult and neonatal monocytes after LPS stimulation using a commercially available annexin V binding assay. Monocytes from cord blood and adult peripheral blood were obtained as outlined above. Isolated monocytes were either labeled immediately with annexin V-FITC or were stimulated for 14 hours with LPS 10 ng/ml prior to labeling (this time point was derived empirically to maximize apoptosis). Annexin V-FITC staining was completed via the Annexin V-FITC Apoptosis Detection Kit I (BD Biosciences, San Jose, CA) using 5 μl of propidium iodine and 5 μl annexin V-FITC as recommended by the manufacturer. Analysis by flow cytometry was accomplished on a FACS Calibur automated benchtop flow cytometer. Data obtained by flow cytometry was analyzed by non-parametric t-test (Mann-Whitney test). An alpha level of 0.05 was considered statistically significant.

### Statistical analysis

Microarrays were normalized and tested for differential expression using methods described previously [18]. Differential expression was concluded if the genes met the following criteria: a minimum expression level at least 10 times above background at one or more time points, a minimum 1.5-fold difference in the mean expression values between groups at one or more time points, and a minimum of 80% reproducibility using the jack-knife method. A jack-knife is the most common type of Leave-one-out-cross-validation (LOOCV); it is used here to cross-validate genes selected by differential analysis [19]. Time series analysis was performed using the hypervariable (HV) gene method previously described by our group [20].

After selection, HV genes are clustered and interrogated for gene-gene interactions. K-means clustering, an unsupervised technique, was performed on the HV genes to create unbiased clusters. Discriminate function analysis (DFA), a supervised technique, was used to determine and spatially map gene-to-gene interactions [21].

All statistical analysis was performed in Matlab R14 (Natick, MA) and Statistica v7 (Tulsa, OK, USA). An alpha level of 0.05 was considered statistically significant for all analyses.

Analysis of the apoptosis assays was undertaken using both parametric and non-parametric analysis methods. Parametric analysis was undertaken using the student's t-test; non-parametic analysis used the Mann-Whitney U-test. A p-value of > 0.05 was the threshold for rejecting the null hypothesis.

#### Discriminant function analysis

DFA is a method that identifies a subset of genes whose expression values can be linearly combined in an equation, denoted a root, whose overall value is distinct for a given characterized group. DFA therefore, allows the genes that maximally discriminate among the distinct groups analyzed to be identified. In the present work, a variant of the classical DFA, named the Forward Stepwise Analysis, was used to select the set of genes whose expression maximally discriminated among experimentally distinct groups. The Forward Stepwise Analysis was built systematically in an iterative manner. Specifically, at each step all variables were reviewed to identify the one that most contributes to the discrimination between groups. This variable was included in the model, and the process proceeded to the next iteration. The statistical significance of discriminative power of each gene was also characterized by partial Wilk's Lambda coefficients, which are equivalent to the partial correlation coefficient generated by multiple regression analyses. The Wilk's Lambda coefficient used a ratio of within-group differences and the sum of within-plus between-group differences. Its value ranged from 1.0 (no discriminatory power) to 0.0 (perfect discriminatory power).

#### Computer analysis of functional associations between differentially expressed genes

In addition to the above analyses, genes showing the most significant differences between neonatal and adult cells were characterized functionally using pre-existing databases such as PubMed, BIND, KEGG, and Ontoexpress. Biological associations of the differentially expressed genes were modelled using Ingenuity Pathways Analysis (Redwood City, CA). Data analyzed through this technique can then be resolved into cogent models of the specific biological pathways activated under the experimental conditions used in the microarray analyses.

## Results

### Differential gene expression analysis

**Figure 1:**
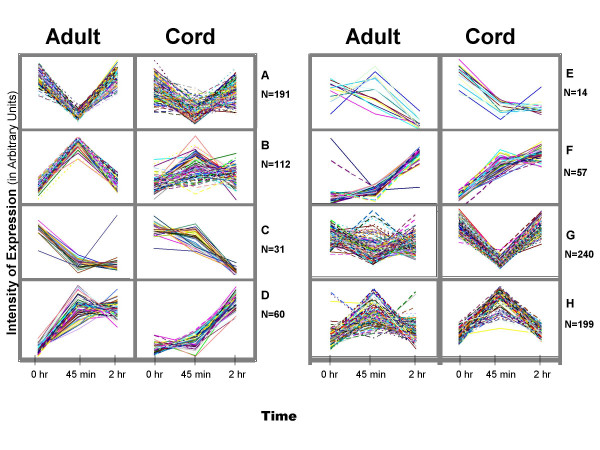
LPS-stimulated genes in cord blood and adult monocytes can be differentiated on the basis of kinetics of expression. Expression level (in relative intensity units) is shown of the y-axis and time on the x-axis. At the 45 min time point, significant differences in expression level were seen between adult and neonatal monocytes for each of the gene groups A-H.

**Figure 2:**
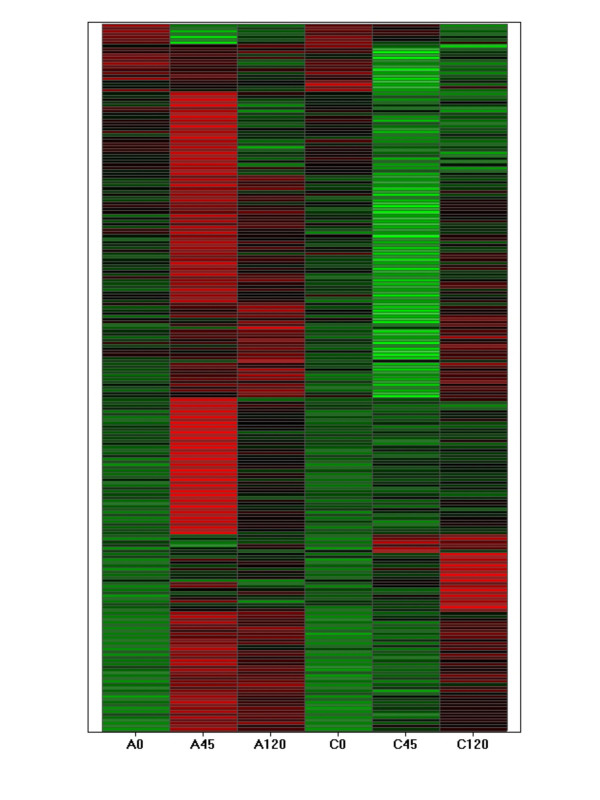
Heat map representation of differences in gene expression of adult and cord blood monocytes in response to LPS. Z-transformed scores of the mean expression values for adult monocytes prior to (A0), after 45 min (A45), and after 120 min (A120) of LPS exposure are graphically shown to the left. Similar scores from cord blood monocytes prior to (C0), after 45 min (C45), and after 120 min C120) of LPS exposure, respectively. The heat map was produced using software from Spotfire Decision Site (Somerville, MA).

Table 1 lists genes determined to be differentially expressed between cord and adult peripheral blood monocytes, as described above. No genes were found to be statistically significantly differentially expressed between adult and cord monocytes in the absence of LPS exposure. 168 genes were differentially expressed between adult and cord monocytes after 45 min incubation with LPS. 95% of these genes (159 of 168) were over-expressed in adult relative to cord monocytes. After 120 minutes of LPS exposure, 24 genes were differentially expressed between adult and cord monocytes. Of the latter genes, 23 were more highly expressed in cord than adult monocytes. This pattern of differentially expressed genes suggested an initial delayed response to LPS followed by an enhanced transcription of genes in cord relative to adult monocytes. To test this hypothesis, k-means clustering was used to categorize differentially expressed genes based on their temporal profiles. Relative decreases in gene transcription by cord monocytes at 45 min were seen in 6 of the 9 clusters (Figure 1). Each of these clusters contained between 15 and 46 genes. Examination of the clusters showed that differences between groups after 45 minutes of LPS exposure were attributable to a) genes in certain clusters that were up-regulated in adult monocytes only, b) genes in other clusters that were down-regulated in cord monocytes only, or c) genes in yet other clusters that were up-regulated in adult and down-regulated in cord monocytes. These results, summarized in a heat map in Figure 2, indicated a high complexity of gene expression differences between adult monocytes and cord blood monocytes in response to LPS.

**Table 1:** Differentially expressed genes between adult and cord monocytes at specific time points. T = time (min) at which the sample was taken. Numbers indicate corrected expression values.

				**Adult**	**Adult**	**Adult**	**Cord**	**Cord**	
	**Genbank #**	**Symbol**	**Gene Description**	**T = 0**	**t = 45**	**t = 120**	**t = 0**	**t = 45**	**t = 120**
**Apoptosis**									
	NM_033423	CTLA1	Similar to granzyme B (granzyme 2, cytotoxic T-lymphocyte-associated serine esterase 1)	317	**419**	299	199	**193**	264
	AB037796	PDCD6IP	Programmed cell death 6 interacting protein	75	**155**	68	79	**70**	81
	NM_024969	TAIP-2	TGFb-induced apoptosis protein 2	63	**113**	107	53	**68**	116
	NM_003127	SPTAN1	Spectrin, alpha, non-erythrocytic 1 (alpha-fodrin)	713	842	***1171***	724	824	***2093***
**Protein synthesis, processing, degradation**									
	AK001313	RPLP0	Ribosomal protein, large, P0	704	**1465**	947	703	**756**	669
	NM_006799	PRSS21	Protease, serine, 21 (testisin)	204	**789**	457	169	**360**	400
	NM_003774	GALNT4	UDP-N-acetyl-alpha-D-galactosamine:polypeptide N-acetylgalactosaminyltransferase 4 (GalNAc-T4)	576	**651**	648	528	**378**	578
	AK057790		cDNA FLJ25061 fis, clone CBL04730	245	**373**	302	244	**215**	200
	NM_004223	UBE2L6	Ubiquitin-conjugating enzyme E2L 6	128	**191**	146	108	**99**	109
	NM_014710	GPRASP1	KIAA0443 gene product	122	**182**	106	113	**119**	95
	NM_021090	MTMR3	Myotubularin related protein 3	109	**171**	137	108	**87**	138
	AF339824	HS6ST3	Heparan sulfate 6-O-sulfotransferase 3	89	**112**	91	94	**46**	76
	NM_012180	FBXO8	F-box only protein 8	40	**67**	42	45	**33**	43
	U66589	RPL5	Ribosomal protein L5	34	**48**	37	30	**26**	36
	NM_001870	CPA3	Carboxypeptidase A3 (mast cell)	183	***495***	610	146	***949***	756
	NM_006145	DNAJB1	DnaJ (Hsp40) homolog, subfmaily B, member 1	179	277	***408***	168	299	***745***
	AK025547	MRPL30	Mitochondrial ribosomal protein L30	83	118	***126***	81	101	***211***
	NM_000439	PCSK1	Proprotein convertase subtilisin/kexin type 1	39	55	***53***	40	78	***88***
**Cell/Organism Movement**									
	NM_002067	GNA11	Guanine nucleotide binding protein (G protein), alpha 11 (Gq class)	555	**870**	607	540	**468**	664
	NM_002465	MYBPC1	Myosin binding protein C, slow type	81	**140**	154	88	**80**	161
	NM_003275	TMOD	Tropomodulin	276	***151***	481	257	***344***	503
	AK026164	MYL6	Myosin, light polypeptide 6, alkali, smooth muscle and non-muscle	7	***6***	48	5	***16***	11
**Small Molecule Interactions**									
	NM_006030	CACNA2D2	Calcium channel, voltage-dependent, alpha 2/delta subunit 2	670	**1390**	1021	641	**639**	946
	AK025170	SFXN5	FLJ21517 fis, clone COL05829	431	**537**	437	405	**295**	374
	NM_021097	SLC8A1	Solute carrier family 8 (sodium/calcium exchanger), member 1	396	**456**	458	412	**276**	369
**Signal Transduction**									
	NM_032144	RAB6C	RAB6C	827	**1658**	1307	626	**773**	1251
	NM_001982	ERBB3	V-erb-b2 erythroblastic leukemia viral oncogene homolog 3	603	**1375**	671	555	**584**	643
	AK026479	SNX14	Sorting nexin 14	682	**1207**	879	624	**567**	883
	NM_018979	PRKWNK1	Protein kinase, lysine deficient 1	451	**813**	782	516	**480**	792
	NM_004811	LPXN	Leupaxin	329	**539**	445	323	**298**	503
	BC005365		clone IMAGE:3829438, mRNA, partial cds	257	**418**	275	275	**275**	206
	NM_004723	ARHGEF2	Rho/rac guanine nucleotide exchange factor (GEF) 2	215	**300**	228	197	**176**	186
	AF130093	MAP3K4	Mitogen-activated protein kinase kinase kinase 4	237	**285**	275	221	**171**	223
	AK000383	MKPX	Mitogen-activated protein kinase phosphatase x	218	**221**	244	233	**126**	197
	NM_022304	HRH2	Histamine receptor H2	45	**121**	86	42	**74**	79
	NM_030753	WNT3	Wingless-type MMTV integration site family member 3	105	**117**	92	109	**63**	81
	AB024574	GTPBP2	GTP binding protein 2	89	**90**	99	74	**57**	92
	NM_002836	PTPRA	Protein tyrosine phosphatase, receptor type, A	8	***6***	80	6	***16***	28
	NM_003656	CAMK1	Calcium/calmodulin-dependent protein kinase I	4940	10131	***4446***	4785	4907	***7190***
**Cellular Metabolism & Cell Division**									
	NM_006170	NOL1	Nucleolar protein 1 (120 kD)	575	**1815**	1021	499	**896**	1093
	AL133115	COVA1	Cytosolic ovarian carcinoma antigen 1	1381	**1294**	848	1309	**658**	808
	D86962	GRB10	Growth factor receptor-bound protein 10	619	**906**	200	609	**512**	179
	NM_005628	SLC1A5	Solute carrier family 1 (neutral amino acid transporter), member 5	338	**801**	600	311	**397**	524
	D17525	MASP1	Mannan-binding lectin serine protease 1 (C4/C2 activating component of Ra-reactive factor)	372	**654**	43	361	**325**	55
	NM_016518	PIPOX	Pipecolic acid oxidase	240	**545**	330	221	**293**	286
	NM_012157	FBXL2	F-box and leucine-rich repeat protein 2	274	**501**	374	249	**277**	298
	NM_018446	AD-017	Glycosyltransferase AD-017	301	**369**	337	288	**223**	327
	NM_001609	ACADSB	Acyl-Coenzyme A dehydrogenase, short/branched chain	354	**368**	325	273	**211**	276
	NM_001647	APOD	Apolipoprotein D	259	**358**	289	261	**202**	205
	NM_012113	CA14	Carbonic anhydrase XIV	218	**356**	279	251	**194**	270
	AB067472	DKFZP434L1435	KIAA1885 protein	150	**213**	186	166	**119**	163
	NM_002916	RFC4	Replication factor C (activator 1) 4 (37 kD)	102	**177**	119	105	**86**	132
	NM_004889	ATP5J2	ATP synthase, H+ transporting, mitochondrial F0 complex, subunit f, isoform 2	106	**147**	76	102	**76**	62
	AK057066		cDNA FLJ32504 fis, clone SMINT1000016, weakly similar to 2-hydroxyacylsphingosine 1b	69	**121**	126	64	**75**	84
	AK021722	AGPAT5	Lysophosphatidic acid acyltransferase, epsilon	37	**71**	48	42	**39**	46
	NM_003664	AP3B1	Adaptor-related protein complex 3, beta 1 subunit	34	**52**	29	37	**24**	30
	AF146760	Sept10	Septin 10	22	**36**	23	26	**16**	28
	NM_004910	PITPNM	Phosphatidylinositol transfer protein, membrane-associated	2611	***2809***	2410	2974	***4590***	2675
	NM_018216	FLJ10782	Pantothenic acid kinase	10	***9***	10	9	***18***	15
	NM_001714	BICD1	Bicaudal D homolog 1 (Drosophila)	230	562	***407***	197	447	***691***
	AK054944	LENG5	Leukocyte receptor cluster (LRC) member 5	67	100	***91***	78	74	***158***
**Gene Expression**									
	NM_005088	DXYS155E	DNA segment on chromosome X and Y (unique) 155 expressed sequence	4857	**3489**	3214	5177	**2241**	2725
	NM_006298	ZNF192	Zinc finger protein 192	552	**988**	761	537	**578**	820
	NM_004991	MDS1	Myelodysplasia syndrome 1	401	**691**	480	390	**361**	420
	NM_021784	HNF3B	Hepatocyte nuclear factor 3, beta	320	**632**	367	347	**361**	391
	AF153201	LOC58502	C2H2 (Kruppel-type) zinc finger protein	288	**532**	335	244	**297**	324
	NM_025212	IDAX	Dvl-binding protein IDAX (inhibition of the Dvl and Axin complex)	297	**490**	311	303	**254**	241
	AK022962	PBX1	Pre-B-cell leukemia transcription factor 1	237	**456**	326	245	**261**	345
	NM_017617	NOTCH1	Notch-1 homolog	309	**358**	353	324	**208**	370
	NM_001451	FOXF1	Forkhead box F1	165	**347**	306	177	**208**	328
	NM_007136	ZNF80	Zinc finger protein 80 (pT17)	199	**269**	203	205	**143**	177
	NM_021975	RELA	V-rel reticuloendotheliosis viral oncogene homolog A, nuclear factor of kappa light polypeptide gene	184	**221**	139	150	**124**	122
	NM_031214	TARDBP	TAR DNA binding protein	76	**154**	109	74	**91**	90
	NM_014007	ZNF297B	Zinc finger protein 297B	109	**137**	122	109	**77**	111
	NM_014938	MONDOA	Mlx interactor	74	**90**	92	69	**53**	86
	NM_005822	DSCR1L1	Down syndrome critical region gene 1-like 1	45	**80**	30	40	**27**	26
	NM_004289	NFE2L3	Nuclear factor (erythroid-derived 2)-like 3	73	**63**	41	64	**39**	38
	NM_054023	SCGB3A2	Secretoglobin family 3a, member 2	37	**59**	45	43	**34**	49
	NM_012107	BP75	Bromodomain containing protein 75 kDa human homolog	44	**51**	34	37	**22**	30
	NM_007212	RNF2	Ring finger protein 2	48	**40**	30	45	**18**	26
	D89859	ZFP161	Zinc finger protein 161 homolog (mouse)	500	596	***4280***	458	481	***6699***
	NM_014335	CRI1	CREBBP/EP300 inhibitory protein 1	52	84	***86***	57	72	***196***
**Immune Function**									
	NM_014889	MP1	Metalloprotease 1 (pitrilysin family)	352	**401**	398	379	**260**	351
	NM_014312	CTXL	Cortical thymocyte receptor (X. laevis CTX) like	386	**370**	375	392	**224**	299
	NM_002053	GBP1	Guanylate binding protein 1, interferon-inducible, 67 kD	259	**369**	334	245	**214**	251
	NM_005356	LCK	Lymphocyte-specific protein tyrosine kinase	186	**206**	187	235	**124**	181
	NM_000564	IL5RA	Interleukin 5 receptor, alpha	112	**106**	124	121	**63**	150
	NM_001311	CRIP1	Cysteine-rich protein 1 (intestinal)	45	***31***	39	49	***60***	43
	NM_002984	SCYA4	Small inducible cytokine A4 MIP1B	492	2001	***2483***	517	1523	***3897***
	NM_002983	SCYA3	Small inducible cytokine A3 MIP1A	248	1798	***2207***	185	1364	***3673***
	NM_014443	IL17B	Interleukin 17B	663	696	***681***	706	703	***1155***
	NM_006018	HM74	Putative chemokine receptor-GTP-binding protein	13	25	***19***	15	26	***34***
**Miscellaneous Functions**									
	AB033041	VANGL2	Vang, van gogh-like 2 (Drosophila)	983	**1246**	1351	981	**796**	1304
	AK021444	POSTN	Periostin, osteoblast specific factor	569	**917**	789	522	**479**	629
	NM_003691	STK16	Serine/threonine kinase 16	403	**777**	458	395	**348**	393
	NM_006438	COLEC10	Collectin sub-family member 10 (C-type lectin)	284	**762**	500	260	**351**	528
	AK057699		FLJ33137 fis, clone UTERU1000077	375	**637**	613	369	**392**	616
	NM_017671	C20orf42	Chromosome 20 open reading frame 42	362	**557**	551	280	**323**	478
	AK054683	DCLRE1C	DNA cross-link repair 1C	486	**555**	574	476	**293**	515
	NM_033060	KAP4.10	Keratin associated protein 4.10	210	**245**	197	154	**123**	172
	AF319045	CNTNAP2	Contactin associated protein-like 2	112	**215**	173	120	**113**	176
	NM_001046	SLC12A2	Solute carrier family 12 (sodium/potassium/chloride transporters), member 2	158	**148**	184	146	**86**	161
	NM_016279	CDH9	Cadherin 9, type 2 (T1-cadherin)	77	**112**	69	65	**51**	64
	NM_014208	DSPP	Dentin sialophosphoprotein	60	**90**	64	57	**53**	59
	NM_015669	PCDHB5	Protocadherin beta 5	92	**83**	62	98	**42**	47
	AK023198	OPRK1	Opioid receptor, kappa 1	58	**76**	41	48	**46**	38
	NM_018240	KIRREL	Kin of IRRE like (Drosophila)	60	**75**	47	66	**43**	46
	AK056781	ROCK1	Rho-associated, coiled-coil containing protein kinase 1	54	**62**	42	47	**41**	42
	NM_022123	NPAS3	Basic-helix-loop-helix-PAS protein	17	**22**	9	16	**12**	13
	NM_001246	ENTPD2	Ectonucleoside triphosphate diphosphohydrolase 2	3438	3272	***3731***	3767	3590	***6309***
**Unknown Function**									
	AK056884		FLJ32322 fis, clone PROST2003577	2007	**2878**	2008	1825	**1548**	1958
	NM_017812	FLJ20420	Coiled-coil-helix-coiled-coil-helix domain containing 3	1105	**1915**	1370	1125	**940**	1358
	AJ420459	LOC51184	Protein x 0004	661	**1579**	881	603	**771**	768
	BC011575		Similar to RIKEN cDNA 0610031J06 gene, clone IMAGE:4639306	974	**1556**	1412	1020	**844**	1261
	AK057357	FLJ32926	DKFZp434D2472	1188	**1378**	1159	1043	**515**	1136
	NM_025019	TUBA4	tubulin, alpha 4	1446	**1173**	1330	1477	**782**	1366
	AK023150		FLJ13088 fis, clone NT2RP3002102	798	**1087**	905	845	**564**	785
	NM_017833	C21orf55	Chromosome 21 open reading frame 55	741	**1079**	799	687	**508**	665
	BC001407		Similar to cytochrome c-like antigen	524	**1004**	629	506	**502**	577
	AK023104		FLJ22648 fis, clone HSI07329	441	**984**	621	488	**471**	495
	AK024617		FLJ20964 fis, clone ADSH00902	824	**955**	745	788	**535**	824
	BC009536		IMAGE:3892368	553	**924**	775	597	**498**	671
	AK056287		FLJ31725 fis, clone NT2RI2006716	435	**862**	907	405	**459**	893
	AK021611		FLJ11549 fis, clone HEMBA1002968	535	**812**	675	545	**392**	630
	BC015119		IMAGE:3951139	445	**784**	487	455	**435**	439
	AK056492		FLJ31930 fis, clone NT2RP7006162	252	**651**	525	266	**367**	457
	AB058711	KIAA1808	KIAA1808 protein	208	**637**	357	199	**339**	366
	BC011266		IMAGE:4156795	354	**632**	432	356	**328**	460
	AK023316		FLJ13254 fis, clone OVARC1000787	416	**596**	357	400	**290**	352
	NM_024696	FLJ23058	Hypothetical protein FLJ23058	456	**541**	346	436	**313**	359
	AF253316		Pheromone receptor (PHRET) pseudogene	136	**520**	425	128	**301**	347
	AK056007	BICD1	Bicaudal D homolog 1 (Drosophila)	704	**505**	439	624	**243**	305
	AB020632	KIAA0825	KIAA0825 protein	249	**498**	353	246	**272**	339
	NM_017609	DKFZp434A1721	Hypothetical protein DKFZp434A1721	182	**485**	319	190	**298**	304
	NM_018190	FLJ10715	Hypothetical protein FLJ10715	202	**483**	310	174	**206**	266
	AK057046		FLJ32484 fis, clone SKNMC2001555	229	**473**	294	261	**302**	228
	NM_013395	AD013	Proteinx0008	448	**461**	496	403	**304**	378
	BC008501	MGC14839	Similar to RIKEN cDNA 2310030G06	379	**414**	329	443	**264**	290
	AK021988		FLJ11926 fis, clone HEMBB1000374	321	**411**	399	280	**218**	288
	AF119872		PRO2272	257	**405**	327	257	**205**	250
	NM_022744	FLJ13868	Hypothetical protein FLJ13868	267	**376**	239	270	**212**	172
	AK022364		FLJ12302 fis, clone MAMMA1001864	172	**355**	316	164	**184**	332
	BC002644	MGC4859	Hypothetical protein MGC4859 similar to HSPA8	282	**335**	382	257	**223**	331
	AK022201		FLJ12139 fis, clone MAMMA1000339	267	**302**	152	235	**123**	131
	NM_017953	FLJ20729	Hypothetical protein FLJ20729	170	**290**	258	138	**170**	218
	AK057473		FLJ32911 fis, clone TESTI2006210	160	**268**	265	163	**123**	247
	U50383	RAI15	Retinoic acid induced 15	206	**265**	236	198	**159**	186
	AK027027		FLJ23374 fis, clone HEP16126	134	**261**	170	134	**152**	141
	AK057288		FLJ32726 fis, clone TESTI2000981	206	**249**	312	216	**152**	244
	U79280	PIPPIN	Ortholog of rat pippin	274	**229**	189	238	**117**	134
	AK023628		FLJ13566 fis, clone PLACE1008330	140	**195**	230	133	**128**	193
	NM_025263	CAT56	CAT56 protein	126	**194**	147	127	**101**	130
	AF311324		Ubiquitin-like fusion protein	191	**189**	179	190	**106**	138
	NM_005708	GPC6	Glypican 6	107	**185**	144	109	**88**	146
	AB037778	KIAA1357	KIAA1357 protein	153	**180**	156	149	**118**	146
	AK055939		FLJ31377 fis, clone NESOP1000087	152	**167**	179	136	**105**	173
	NM_018316	FLJ11078	Hypothetical protein FLJ11078	89	**145**	118	73	**94**	103
	AF402776	BIC	BIC noncoding mRNA	82	**136**	171	96	**88**	153
	BC003416		IMAGE:3450973	64	**133**	93	83	**73**	111
	AL137491		DKFZp434P1530	62	**130**	88	57	**72**	74
	AK057770		FLJ25041 fis, clone CBL03194	110	**130**	114	108	**83**	84
	AB058769	KIAA1866	KIAA1866 protein	89	**126**	122	102	**83**	91
	AB058747	WAC	WW domain-containing adapter with a coiled-coil region	60	**124**	103	57	**76**	77
	AK054885	C6orf31	Chromosome 6 open reading frame 31	51	**119**	108	41	**68**	119
	AK022235		FLJ12173 fis, clone MAMMA1000696	109	**103**	94	90	**62**	77
	AK026853	AOAH	Acyloxyacyl hydrolase (neutrophil)	59	**98**	64	59	**61**	56
	AK024877		FLJ21224 fis, clone COL00694	53	**96**	110	55	**54**	103
	NM_003171	SUPV3L1	Suppressor of var1, 3-like 1 (S. cerevisiae)	65	**93**	60	60	**55**	58
	NM_052933	TSGA13	Testis specific, 13	66	**80**	70	68	**44**	71
	AK057907		FLJ25178 fis, clone CBR09176	42	**77**	31	47	**43**	41
	AK055748		FLJ31186 fis, clone KIDNE2000335	88	**67**	68	79	**44**	71
	BC013757		IMAGE:4525041	40	**54**	39	43	**33**	32
	AL365511		Novel human gene mapping to chomosome 22	19	**48**	29	20	**27**	37
	AK026889	APRIN	Androgen-induced proliferation inhibitor	31	**35**	42	34	**21**	34
	AK057423		FLJ32861 fis, clone TESTI2003589	36	**32**	34	30	**18**	31
	AK055543	MLSTD1	Male sterility domain containing 1	31	**31**	32	27	**18**	30
	AK056513		FLJ31951 fis, clone NT2RP7007177	33	**29**	20	22	**13**	20
	NM_013319	TERE1	Transitional epithelia response protein	22	**28**	19	24	**17**	22
	AK026456		FLJ22803 fis, clone KAIA2685	15	**26**	14	16	**13**	17
	AK021610		cDNA FLJ11548 fis, clone HEMBA1002944	34	**26**	29	31	**15**	28
	AK026823		FLJ23170 fis, clone LNG09984	15	**22**	14	19	**8**	18
	AK056805		FLJ32243 fis, clone PROST1000039	400	***177***	186	343	***314***	160
	NM_012238	SIRT1	Sirtuin silent mating type information regulation 2 homolog 1 (S. cerevisiae)	149	156	**170**	178	134	**109**
	NM_016099	GOLGA7	golgi autoantigen, golgin subfamily a, 7	10493	15165	***9882***	11947	11564	***15698***
	AK022482		FLJ12420 fis, clone MAMMA1003049	6052	9099	***5803***	6362	7620	***9309***
	AK026490	RAB32	RAB32, member RAS oncogene family	3677	7044	***4641***	3671	5553	***7561***
	NM_020684	NPD007	NPD007 protein	674	794	***764***	630	720	***1215***
	AL390158	ATXN7L3	Ataxin 7-like 3	319	460	***378***	339	403	***598***
	NM_017752	FLJ20298	Hypothetical protein FLJ20298	146	237	***282***	133	233	***493***
	AB037743	KIAA1322	KIAA1322 protein	236	202	***199***	239	246	***319***
	AF339819		clone IMAGE:38177	77	111	***110***	96	125	***174***
	AK055215		FLJ30653 fis, clone DFNES2000143	47	48	***58***	43	80	***92***

In addition to the above genes which differed in expression between groups following LPS exposure, 516 genes were also identified that were differentially expressed over time within a group. A supplementary table containing these data is available upon request. For these genes, a similar pattern of dynamic expression was seen as was observed in the other group. Therefore, these genes reflect common responses to LPS in monocytes from both sources.

A subset of genes that were differentially expressed either between adult and cord blood monocytes were selected for validation using the quantitative real-time polymerase chain reaction method (QRT-PCR). These included four genes that differed between groups after 45 min of LPS exposure (*Erbb3, Tmod, Dscr1l1*, and *Sp1*), and six genes that differed in expression after 2 hours of LPS exposure (*Scya4, Gro2, Cri1, Scya3, Scya3l1*, and *Il-1a*). Nine of the ten genes tested for QRT-PCR validation demonstrated similar levels of relative expression in QRT-PCR experiments as in the microarrays. Only CRI1 failed to corroborate the microarray data.

### Hypervariable gene analysis

**Figure 3:**
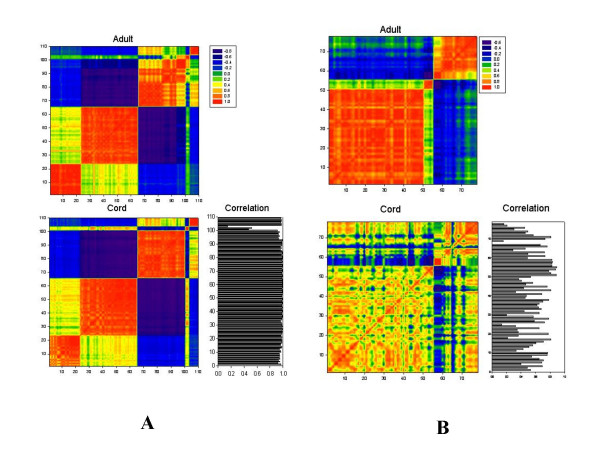
Correlative mosaic for genes selected as HV-genes in cord blood and adult monocytes, belonging to five clusters of highest content. A. Genes of the same cluster in cord and adult; B. Genes of different cluster in cord and adult. Correlation coefficients are color-coded according to the key in the upper right. The correlation between the adult and cord blood monocyte profiles for each gene are shown in black and white, lower right.

One hundred eighty-eight hypervariable (HV) genes were selected from expressed genes in adult and cord blood monocytes based on their changes across three time points. These genes exhibited significantly higher expression variation over time than the majority of genes. Differences in variation between two experimental sample sets, in this case adult and neonatal samples, can represent differences in homeostatic control mechanisms between these two sets [20]. The selected genes were hypervariable in both sample groups. HV genes with highly correlated expression levels in a given population are likely to share function [20]. A correlation based clustering procedure was carried out for these HV genes as described in the methods section. Genes belonging to the 5 largest clusters were used for creation of a graphical output, denoted a correlation mosaic. A correlation mosaic allows identification of the genes within clusters by visual inspection and subsequent functional analysis of genes within clusters (Figures 3A &3B). Figure 3A represents 110 genes of the same cluster allocation between adult and cord blood monocyte samples, demonstrating a very high similarity between cells from these two groups, as measured by the correlation coefficients between genes from adult and cord monocytes with value > 0.90 (figure 3A, black and white graph to the right). Three genes on this list (#101–103) were the exception: transcriptional regulator interacting with the PHS-bromodomain 2 (*Trip-Br2*), interleukin 1 beta (*Il1b*), and the GRO2 oncogene(*Gro2*). These genes may play a critical role in differentiation between adult and cord monocyte behaviour [22,23]. The high similarity of these mosaics presents evidence for the presence of fundamental processes in monocyte development that appear to be quite similar in both groups of samples. The details of the genes used in Figure 3A are presented as Table 2. Another group of 78 genes were found that have different cluster designations between adult and cord blood monocytes (Figure 3B). Details of these genes are listed in Table 3.

**Table 2:** Genes from which correlation mosaics in Figure 3A were derived. Genes in this table show the highest level of correlation by DFA analysis comparing adult and cord blood monocytes.

**Order in mosaic**	**Accession No.**	**Gene symbol**	**Description**
1	NM_017614	BHMT2	Betaine-homocysteine methyltransferase 2
2	NM_001651	AQP5	Aquaporin 5
3	NM_020163	LOC56920	Semaphorin sem2
4	NM_012343	NNT	Nicotinamide nucleotide transhydrogenase
5	NM_000096	CP	Ceruloplasmin (ferroxidase)
6	NM_005819	STX6	Syntaxin 6
7	NM_052951	C20orf167	Chromosome 20 open reading frame 167
8	NM_001348	DAPK3	Death-associated protein kinase 3
9	X73502	KRT20	Cytokeratin 20
10	NM_052887	TIRAP	Toll-interleukin 1 receptor (TIR) domain-containing adapter protein
11	NM_019555	ARHGEF3	Rho guanine nucleotide exchange factor (GEF) 3
12	NM_014380	NGFRAP1	Nerve growth factor receptor (TNFRSF16) associated protein 1
13	NM_001272	CHD3	Chromodomain helicase DNA binding protein 3
14	NM_005842	SPRY2	Sprouty homolog 2 (Drosophila)
15	NM_012332	MT-ACT48	Mitochondrial Acyl-CoA Thioesterase
16	BC015041	VATI	Vesicle amine transport protein 1
17	NM_003872	NRP2	Neuropilin 2
18	NM_005849	IGSF6	Immunoglobulin superfamily, member 6
19	NM_014323	ZNF278	Zinc finger protein 278
20	NM_030674	SLC38A1	Solute carrier family 38, member 1
21	NM_004153	ORC1L	Origin recognition complex, subunit 1-like (yeast)
22	NM_005249	FOXG1B	Forkhead box G1B
23	NM_021048	MAGEA10	Melanoma antigen, family A, 10
24	M60502	FLG	Filaggrin
25	NM_004997	MYBPH	Myosin binding protein H
26	J05046	INSRR	Insulin receptor-related receptor
27	M33987	CA1	Carbonic anhydrase I
28	D31886	RAB3GAP	RAB3 GTPase-ACTIVATING PROTEIN
29	L24498	GADD45A	Growth arrest and DNA-damage-inducible, alpha
30	L07590	PPP2R3	Protein phosphatase 2 (formerly 2A), regulatory subunit B" (PR 72), alpha isoform and (PR 130), bet
31	D87024	IGLV4-3	Immunoglobulin lambda variable 4-3
32	L35848	MS4A3	Membrane-spanning 4-domains, subfamily A, member 3 (hematopoietic cell-specific)
33	M18216	CEACAM6	Carcinoembryonic antigen-related cell adhesion molecule 6 (non-specific cross reacting antigen)
34	M11952	TRBV7–8	T cell receptor beta variable 7–8
35	D89094	PDE5A	Phosphodiesterase 5A, cGMP-specific
36	M77140	GAL	Galanin
37	D13628	ANGPT1	Angiopoietin 1
38	M81635	EPB72	Erythrocyte membrane protein band 7.2 (stomatin)
39	D89859	ZFP161	Zinc finger protein 161 homolog (mouse)
40	D26069	CENTB2	Centaurin, beta 2
41	L10717	ITK	IL2-inducible T-cell kinase
42	L04282	ZNF148	Zinc finger protein 148 (pHZ-52)
43	L41944	IFNAR2	Interferon (alpha, beta and omega) receptor 2
44	M82882	ELF1	E74-like factor 1 (ets domain transcription factor)
45	L26339	RCD-8	Autoantigen
46	D87328	HLCS	Holocarboxylase synthetase (biotin-[proprionyl-Coenzyme A-carboxylase (ATP-hydrolysing)] ligase)
47	D00943	MYH6	Myosin, heavy polypeptide 6, cardiac muscle, alpha (cardiomyopathy, hypertrophic 1)
48	D00099	ATP1A1	ATPase, Na+/K+ transporting, alpha 1 polypeptide
49	L36531	ITGA8	Integrin, alpha 8
50	D42084	METAP1	Methionyl aminopeptidase 1
51	M76766	GTF2B	General transcription factor IIB
52	J04621	SDC2	Syndecan 2 (heparan sulfate proteoglycan 1, cell surface-associated, fibroglycan)
53	D31888	RCOR	REST corepressor
54	L32832	ATBF1	AT-binding transcription factor 1
55	D86981	APPBP2	Amyloid beta precursor protein (cytoplasmic tail) binding protein 2
56	M94362	LMNB2	Lamin B2
57	M54968	KRAS2	V-Ki-ras2 Kirsten rat sarcoma 2 viral oncogene homolog
58	D42046	DNA2L	DNA2 DNA replication helicase 2-like (yeast)
59	D86964	DOCK2	Dedicator of cyto-kinesis 2
60	D50683	TGFBR2	Transforming growth factor, beta receptor II (70–80 kD)
61	M96843	ID2B	Striated muscle contraction regulatory protein
62	M61906	PIK3R1	Phosphoinositide-3-kinase, regulatory subunit, polypeptide 1 (p85 alpha)
63	M12679	HUMMHCW1A	Cw1 antigen
64	M63623	OMG	Oligodendrocyte myelin glycoprotein
65	J04162	FCGR3B	Fc fragment of IgG, low affinity IIIb, receptor for (CD16)
66	L48516	PON3	Paraoxonase 3
67	M54927	PLP1	Proteolipid protein1 (Pelizaeus-Merzbacher disease, spastic paraplegia 2, uncomplicated)
68	D86973	GCN1L1	GCN1 general control of amino-acid synthesis 1-like 1 (yeast)
69	D43968	RUNX1	Runt-related transcription factor 1 (acute myeloid leukemia 1-aml1 oncogene)
70	L05500	ADCY1	Adenylate cyclase 1 (brain)
71	D80010	LPIN1	Lipin 1
72	D50918	SEPT6	Septin 6
73	D86988	RENT1	Regulator of nonsense transcripts 1
74	M90391	IL16	Interleukin 16 (lymphocyte chemoattractant factor)
75	M62324	MRF-1	Modulator recognition factor I
76	L77565	DGS-H	DiGeorge syndrome gene H
77	D86970	TIAF1	TGFB1-induced anti-apoptotic factor 1
78	D38169	ITPKC	Inositol 1,4,5-trisphosphate 3-kinase C
79	D87684	UBXD2	UBX domain-containing 2
80	D84454	SLC35A2	Solute carrier family 35 (UDP-galactose transporter), member 2
81	M97496	GUCA2A	Guanylate cyclase activator 2A (guanylin)
82	M95585	HLF	Hepatic leukemia factor
83	L38517	IHH	Indian hedgehog homolog (Drosophila)
84	L20860	GP1BB	Glycoprotein Ib (platelet), beta polypeptide
85	M26880	UBC	Ubiquitin C
86	D86962	GRB10	Growth factor receptor-bound protein 10
87	D63481	SCRIB	Scribble
88	D17525	MASP1	Mannan-binding lectin serine protease 1 (C4/C2 activating component of Ra-reactive factor)
89	L26584	RASGRF1	Ras protein-specific guanine nucleotide-releasing factor 1
90	M65066	PRKAR1B	Protein kinase, cAMP-dependent, regulatory, type I, beta
91	J05158	CPN2	Carboxypeptidase N, polypeptide 2, 83 kD
92	L36861	GUCA1A	Guanylate cyclase activator 1A (retina)
93	L11239	GBX1	Gastrulation brain homeo box 1
94	D90145	SCYA3L1	Small inducible cytokine A3-like 1
95	M96739	NHLH1	Nescient helix loop helix 1
96	M12959	TRA@	T cell receptor alpha locus
97	D80005	C9orf10	C9orf10 protein
98	M13231	TRGC2	T cell receptor gamma constant 2
99	D28588	SP2	Sp2 transcription factor
100	M57732	TCF1	Transcription factor 1, hepatic-LF-B1, hepatic nuclear factor (HNF1), albumin proximal factor
101	NM_014755	TRIP-Br2	Transcriptional regulator interacting with the PHS-bromodomain 2
102	NM_000576	IL1B	Interleukin 1, beta
103	NM_002089	GRO2	GRO2 oncogene
104	NM_002089x	GPRC5D	G protein-coupled receptor, family C, group 5, member D
105	NM_002713	PPP1R8	Protein phosphatase 1, regulatory (inhibitor) subunit 8
106	NM_014383	TZFP	Testis zinc finger protein
107	NM_012248	SPS2	Selenophosphate synthetase 2
108	AL137438	SEC15L	SEC15 (S. cerevisiae)-like
109	NM_005387	NUP98	Nucleoporin 98 kD
110	NM_003476	CSRP3	Cysteine and glycine-rich protein 3 (cardiac LIM protein)

**Table 3:** Genes from which the mosaic in Figure 3B were derived. Genes from which correlation mosaics in Figure 3B were derived. Genes in this table show the greatest differences by DFA analysis comparing adult and cord blood monocytes.

**Order in Mosaic**	**Accession No.**	**Gene Symbol**	**Description**
1	AK055855	CLDN10	Claudin 10
2	NM_000565	IL6R	Interleukin 6 receptor
3	NM_006150	LMO6	LIM domain only 6
4	NM_022787	NMNAT	NMN adenylyltransferase-nicotinamide mononucleotide adenylyl transferase
5	NM_002743	PRKCSH	Protein kinase C substrate 80K-H
6	NM_004847	AIF1	Allograft inflammatory factor 1
7	NM_021073	BMP5	Bone morphogenetic protein 5
* 8	AK025306	CLK1	CDC-like kinase 1
9	NM_004280	EEF1E1	Eukaryotic translation elongation factor 1 epsilon 1
* 10	NM_004432	ELAVL2	ELAV (embryonic lethal, abnormal vision, Drosophila)-like 2 (Hu antigen B)
11	NM_012181	FKBP8	FK506 binding protein 8 (38 kD)
12	NM_002091	GRP	Gastrin-releasing peptide
13	NM_016355	LOC51202	Hqp0256 protein
14	NM_021204	MASA	E-1 enzyme
15	NM_004204	PIGQ	Phosphatidylinositol glycan, class Q
16	NM_002928	RGS16	Regulator of G-protein signalling 16
17	NM_005839	SRRM1	Serine/arginine repetitive matrix 1
18	NM_003166	SULT1A3	Sulfotransferase family, cytosolic, 1A, phenol-preferring, member 3
19	NM_000356	TCOF1	Treacher Collins-Franceschetti syndrome 1
20	NM_016437	TUBG2	Tubulin, gamma 2
* 21	NM_022568	ALDH8A1	Aldehyde dehyrdogenase 8 family, member A1
22	AF209930	CHRD	Chordin
23	NM_005274	GNG5	Guanine nucleotide binding protein (G protein), gamma 5
24	NM_018384	IAN4L1	Immune associated nucleotide 4 like 1 (mouse)
25	NM_000640	IL13RA2	Interleukin 13 receptor, alpha 2
26	AK021692	LOC51141	Insulin induced protein 2
27	NM_012443	SPAG6	Sperm associated antigen 6
28	NM_003155	STC1	Stanniocalcin 1
29	NM_022003	FXYD6	FXYD domain-containing ion transport regulator 6
30	NM_002763	PROX1	Prospero-related homeobox 1
31	NM_002836	PTPRA	Protein tyrosine phosphatase, receptor type, A
32	AL136835	TOLLIP	Toll-interacting protein
33	AB058691	ALX4	Aristaless-like homeobox 4
34	AF112345	ITGA10	Integrin, alpha 10
35	NM_022788	P2RY12	Purinergic receptor P2Y, G protein-coupled, 12
36	NM_001213	C1orf1	Chromosome 1 open reading frame 1
37	NM_005860	FSTL3	Follistatin-like 3 (secreted glycoprotein)
38	NM_013320	HCF-2	Host cell factor 2
39	NM_058246	LOC136442	Similar to MRJ gene for a member of the DNAJ protein family
40	NM_020169	LXN	Latexin protein
41	BC008993	MGC17337	Similar to RIKEN cDNA 5730528L13 gene
42	BC002712	MYCN	V-myc myelocytomatosis viral related oncogene, neuroblastoma derived (avian)
43	AK026164	MYL6	Myosin, light polypeptide 6, alkali, smooth muscle and non-muscle
44	NM_006215	SERPINA4	Serine (or cysteine) proteinase inhibitor, clade A (alpha-1 antiproteinase, antitrypsin), member 4
45	NM_004790	SLC22A6	Solute carrier family 22 (organic anion transporter), member 6
46	NM_022911	SLC26A6	Solute carrier family 26, member 6
47	NM_003374	VDAC1	Voltage-dependent anion channel 1
48	NM_017818	WDR8	WD repeat domain 8
49	NM_003416	ZNF7	Zinc finger protein 7 (KOX 4, clone HF.16)
50	NM_002313	ABLIM	Actin binding LIM protein
51	NM_012074	CERD4	Cer-d4 (mouse) homolog
52	NM_000787	DBH	Dopamine beta-hydroxylase (dopamine beta-monooxygenase)
* 53	NM_000561	GSTM1	Glutathione S-transferase M1
54	BC014075	GTPBP1	GTP binding protein 1
55	NM_033260	HFH1	Winged helix/forkhead transcription factor
56	NM_033033	KRTHB2	Keratin, hair, basic, 2
57	NM_004789	LHX2	LIM homeobox protein 2
58	NM_014106	PRO1914	PRO1914 protein
* 59	NM_006799	PRSS21	Protease, serine, 21 (testisin)
* 60	NM_002900	RBP3	Retinol binding protein 3, interstitial
61	NM_033022	RPS24	Ribosomal protein S24
* 62	AB029021	TRIM35	Tripartite motif-containing 35
* 63	NM_020989	CRYGC	Crystallin, gamma C
* 64	BI198124	HMG1L10	High-mobility group (nonhistone chromosomal) protein 1-like 10
65	NM_014163	HSPC073	HSPC073 protein
66	AF181985	JIK	STE20-like kinase
67	NM_017607	PPP1R12C	Protein phosphatase 1, regulatory (inhibitor) subunit 12C
* 68	NM_002873	RAD17	RAD17 homolog (S. pombe)
69	NM_022095	ZNF335	Zinc finger protein 335
* 70	M90355	BTF3L2	Basic transcription factor 3, like 2
71	NM_002079	GOT1	Glutamic-oxaloacetic transaminase 1, soluble (aspartate aminotransferase 1)
72	NM_004146	NDUFB7	NADH dehydrogenase (ubiquinone) 1 beta subcomplex, 7 (18 kD, B18)
73	L38486	MFAP4	Microfibrillar-associated protein 4
* 74	AF111848	ACTB	Actin, beta
75	NM_001916	CYC1	Cytochrome c-1

**Figure 4:**
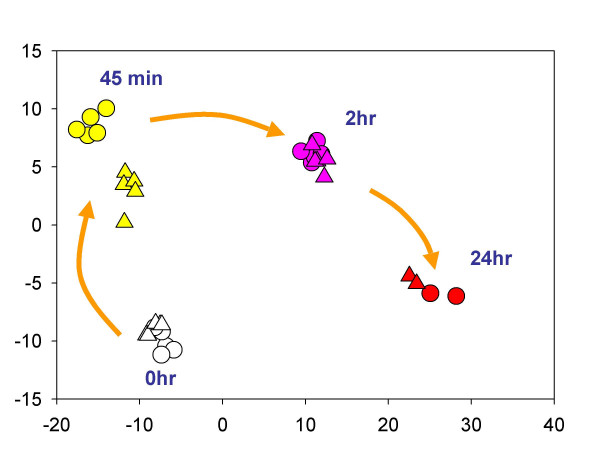
DFA analysis of phases of monocyte activation comparing cord and adult cells. DFA identified a subset of genes (see Table 3) whose expression values can be linearly combined in an equation, denoted a root, whose overall value is distinct for a given characterized group. These roots used as coordinate for presentation of these groups of samples in scatterplot. Results from individual samples for adult monocyte (circles) and cord monocytes (triangles) are discussed in the text. Results from individual samples for adult monocyte (circles) and cord monocytes (triangles) are shown.

We analyzed these genes using DFA in order to find those genes most likely to highlight the differences between cord and adult monocytes. DFA identified genes having high discriminatory capabilities. The DFA software selected genes from Table 3 with highest discriminatory capabilities for this case. A total of 12 genes (marked with asterisk in Table 3) were used by the DFA program to differentiate dynamical changes in both cord and adult monocytes after LPS stimulation. Values of the roots obtained by DFA analysis were used to graphically depict the differences of the gene expression values obtained in cord and adult samples in different stages after stimulation (Fig. 4). The spatial organization of the elements in this representation provides a measure of the overall similarity of the dynamic behaviour of these samples. The greatest temporal changes in gene expression for cord and adult monocytes noted above after 45 min of LPS exposure were also observed in the analysis using these 12 genes. However, almost no differences occurred at the 2 hr time point between cord and adult cells suggesting that the global behavior of the cells is similar, but the kinetics of change differ i.e. many of the changes are the same in both groups, but they occur at different rates.

### Apoptosis assays

The products of a subset of genes that were differentially expressed between groups after 45 min exposure to LPS are involved in apoptosis. We therefore performed a series of functional experiments comparing apoptosis in adult (n = 10) and neonatal (n = 10) cord bloods. Results of these assays are shown in Table 4. Annexin assays demonstrated that adult monocytes display different kinetics for both apoptosis and necrosis as compared with neonatal monocytes. Flow cytometry revealed that 43 ± 5% (mean + SD) of adult and 53 + 8% of neonatal monocytes are undergoing apoptosis after stimulation with LPS for 14 hours (p < 0.002), while 38 + 8% of adult and 25 + 9% of neonatal monocytes are necrotic after 14 hours of LPS stimulation (p < 0.003). The number of live monocytes after 14 hours of LPS stimulation was not statistically different between the two groups. There was also no statistically significant difference in the number of live, apoptotic, or necrotic monocytes between adult and neonatal samples prior to LPS stimulation (data not shown).

**Table 4:** Results of Annexin Binding Assays

**Cell Type**	**Apoptotic Cells**	**Necrotic Cells**	**Significance**
Adult monocytes	43 ± 5%	38 % ± 8%	P < 0.002
Cord blood monocytes	53 ± 8%	25% ± 9%	P < 0.003

## Discussion

Following a given physiologic stimulus, signalling kinase activation, transcription factor translocation, and gene transcription all occur in rapid order. However, like all biological processes, mRNA accumulation (or decreases) does not occur uniformly, and we hypothesized that examining the kinetics of mRNA accumulation or disappearance might provide clues into relevant cellular dynamics. We used a well-developed and validated gene expression microarray to examine the dynamics of mRNA accumulation and differences between adult and neonatal monocytes in that process.

Genes were found to be differentially expressed between adult and cord monocytes after either 45 or 120 minutes of LPS exposure, with little difference at 24 hr (see Figure 4). Interestingly, no statistically significant differences in gene expression were observed between these groups in untreated cells. Previous reports by others indicated altered functions of cord blood monocytes in cytokine secretion and cellular adhesion. Results from this study cast new light on these findings and add complexity to understanding such differences. In some cases, our data support previous speculations about neonatal immune function. For example, the increased expression of IL-17B in neonatal monocytes is consistent with the observations of Vanden Eijnden and colleagues that newborns compensate for their relative immune deficiency by over-expression of the IL23-IL-17 signalling pathway in dendritic cells [24]. Similarly, we found significant elevations in cord monocyte transcripts of the chemokines MIP1B and MIP1A after 2 hrs of LPS exposure, consistent with Sullivan and colleagues' report of higher amounts of MIPα in cord blood samples compared with adults [25]. On the other hand, transcripts for cadherin 9, *Rock1*, periostin, heparin sulfate 6-O-sulfotransferase 3, and C20orf42, whose products participate in various mechanisms that are associated with adhesion [26-28] were statistically significantly increased in adult monocytes after 45 min of LPS exposure, although no differences in expression for these genes between groups were detected at the later time point. These data suggest complex, dynamic relations for genes whose products are associated with cellular adhesion, and collectively highlight the importance of examining gene expression profiles (or related protein expression levels) over time.

The limits of gene expression profiling as a technique, albeit a very useful technique, must be acknowledged. The technique examines only RNA transcripts, not protein synthesis. Thus, alterations in other critical inflammatory mediators, such as eicosanoids, remain unobserved with this method. Furthermore, it is well known that there are many proteins, including critical inflammatory mediators, whose synthesis and secretion is not directly related in mRNA accumulation [29]. Thus, gene expression profiling should be complemented with other methods in order to maximize there potential.

In the final analysis, the utility of gene expression profiling will be demonstrated only if they provide insights into relevant physiologic or pathophysiologic function. For that reason, we elected to test the validity of the array data by examining a physiologic mechanism implicated by computer modelling of the array data. As noted in Table 1, adult monocytes over-expressed a small number of genes associated with the regulation of apoptosis. Since monocyte activation is a "balancing act" between signals inducing apoptosis and those inducing activation and differentiation [30,31], differences in the kinetics of expression or activation of enzymes or transcription factors that regulate apoptosis could have a crucial outcome on whether monocyte responses are pro- or anti-inflammatory. Annexin assays confirmed that there are significant differences in the appearance of apoptotic cells between adults and newborn monocytes (Table 4). Since apoptotic cells dampen the inflammatory response, it is interesting to speculate that the related blunted neonatal response to inflammatory stimuli (including infection) may result, at least in part, from the excessive production of apoptotic cells during monocyte activation.

There has been, to our knowledge, one previously published paper using gene expression arrays to study neonatal monocyte function [14]. Our findings differ somewhat from those described by these authors. The most obvious difference was our finding of no statistically significant differences between adult and cord blood samples in the resting state. We should note, however, that it is otherwise difficult to compare the two studies. Jiang and colleagues used a 1000-fold greater dose of LPS to stimulate the monocytes, and RNA was prepared after 18 hr of stimulation. Thus, it is difficult to determine which of the effects observed by these authors were the direct result of LPS activation or were mediated through autocrine activation by proteins secreted in response to LPS. Furthermore, the non-physiologic dose of LPS used by those authors makes the biological/pathological relevance of that study difficult to interpret. Finally, we should note that the study by Jiang and colleagues used different methodologies for purifying monocytes. While our method, positive selection using CD14-coated microbeads, carries the theoretical risk of activating the cells through TLR-4/CD14 signaling pathways, adherence procedures carry the greater risk of activating the cells, as β2 integrins are activated during the adherence process.

From the bioinformatics standpoint, our data demonstrate how gene microarray experiments can quickly move from the generation of gene lists to the development of plausible and testable models of relevant biology and physiology. Specifically, they demonstrate that computer-assisted, physiologic modelling is another means of corroborating array findings and provides the advantage of providing an approach for immediately testing the biological relevance of microarray data before embarking on the sometimes laborious task of confirming differential expression of dozens or even hundreds of genes identified in a microarray experiment. As described in the results section, the differences between groups in gene expression at 45 min were attributable to a unique up-regulation of specific genes in adult monocytes, a unique down-regulation of other genes in cord monocytes, or a combination of both processes for other genes. We have searched for mechanisms that account for these patterns. Specifically, we have analyzed the genes within derived k-means clusters to determine if a large number of genes within a cluster are related to overlapping functions using Ingenuity Pathway Assist software, or alternatively to shared transcriptional response elements upstream of these genes. However, these strategies have failed to elucidate reasons to explain these findings.

Our studies also suggest that, while expensive and time-consuming to undertake, studying the kinetics of gene expression using microarrays can be highly informative. The previously reported study [14] examining gene expression differences between adult and cord blood monocytes was performed at only a single time point (18 hr after activation with a non-physiologic dose of LPS). Our studies suggest that the relevant biology may lie not in the specific genes that are differentially expressed at one particular time point, but, as one would predict with a dynamic system, which genes are expressed when. Timing of mRNA accumulation could determine, among other things, whether pro-apoptotic signals are processed in monocytes before cellular necrosis ensues.

The validity of the dynamic/kinetic approach is further supported by the correlation analyses (Figures 3 and 4). These analyses demonstrate clearly that the accumulation of a specific mRNA is not an independent event. Gene transcription and mRNA degradation are dynamic processes closely tied to the accumulation or degradation of other mRNAs and the transcription of their cognate proteins. We contend that, without this dynamic view of cellular activity, investigators attempting to use microarray data to elucidate relevant biological or pathological processes will encounter unnecessary obstacles in attempts to move from the generation of gene lists to testing specific hypotheses.

## Abbreviations

LPS – Lipopolysaccharide

DFA – Discriminant function analysis

HV – Hypervariable

## References

[B1] Kobayashi S, Ohnuma K, Uchiyama M, Iino K, Iwata S, Dang NH, Morimoto C (2004). Association of CD26 with CD45RA outside lipid rafts attenuates cord blood T-cell activation. Blood.

[B2] Adkins B, LeClerc C, Marshall-Clark S (2004). Neonatal adaptive immunity comes of age. Nature Rev Immunol.

[B3] Garcia AM, Fadel SA, Cao S, Sarzotti M (2000). T cell immunity in neonates. Immunol Res.

[B4] Zhao Y, Dai ZP, Lv P, Gao XM (2002). Phenotypic and functional analysis of human T lymphocytes in early second- and third-trimester fetuses. Clin Exp Immunol.

[B5] Zola H, Fusco M, Weedon H, Macardle PJ, Ridings J, Roberton DM (1996). Reduced expression of the interleukin-2-receptor gamma chain on cord blood lymphocytes: relationship to functional immaturity of the neonatal immune response. Immunol.

[B6] Schuit KE, Powell DA (1980). Phagocytic dysfunction in monocytes of normal newborn infants. Pediatrics.

[B7] Tan ND, Davidson D (1995). Comparative differences and combined effects of interleukin-8, leukotriene B_4_, and platelet-activating factor on neutrophil chemotaxis of the newborn. Pediatr Res.

[B8] Anderson DC, Hughes BJ, Smith CW (1981). Abnormal mobility of neonatal polymorphonuclear leukocytes. J Clin Invest.

[B9] Anderson DC, Freeman KLB, Heerdt B, Hughes BJ, Jack RM, Smith CW (1987). Abnormal stimulated adherence of neonatal granulocytes: impaired induction of surface Mac-1 by chemotactic factors or secretagogues. Blood.

[B10] Torok C, Lundahl J, Hed J, Lagercrantz H (1993). Diversity of regulation of adhesion molecules (Mac-1 and L-selectin) in monocytes and neutrophils from neonates and adults. Arch Dis Child.

[B11] Hariharan D, Ho W, Cutilli J, Campbell DE, Douglas SD (2000). C-C chemokine profile of cord blood mononuclear cells: selective defect in RANTES production. Blood.

[B12] Bessler H, Mendel C, Straussberg R, Gurary N, Aloni D, Sirota L (1999). Effects of dexamethasone on IL-1beta, IL-6, and TNF-alpha production by mononuclear cells of newborns and adults. Biol Neonate.

[B13] Kotiranta-Ainamo A, Rautonen J, Rautonen N (1997). Interleukin-10 production by cord blood mononuclear cells. Pediatric Res.

[B14] Jiang H, Van de Ven C, Satwani P, Baxi LV, Cairo MS (2004). Differential gene expression patterns by oligonucleotide microarray of basal versus lipopolysaccharide-activated monocytes from cord blood versus adult peripheral blood. J Immunol.

[B15] Hirschfeld M, Ma Y, Weis JH, Vogel SN, Weis JJ (2000). Cutting edge: repurification of lipopolysaccharide eliminates signaling through both human and murine toll-like receptor 2. J Immunol.

[B16] Bustin SA (2002). Quantification of mRNA using real-time reverse transcription PCR (RT-PCR): trends and problems. J Endocrinol.

[B17] Tricarico C, Pinzani P, Bianchi S, Paglierani M, Distante V, Pazzagli M, Busin SA, Orlando C (2002). Quantitative real-time reverse transcription polymerase chain reaction: normalisation to rRNA or single housekeeping genes is inappropriate for human tissue biopsies. Analytical Biochem.

[B18] Knowlton N, Dozmorov IM, Centola M (2004). Microarray Data Analysis Toolbox (MDAT): for normalization, adjustment and analysis of gene expression data. Bioinformatics.

[B19] Efron B, Gong G (1983). A leisurely look at the bootstrap, the jackknife, and cross-validation. American Statistician.

[B20] Dozmorov I, Knowlton N, Tang Y, Shields A, Pathipvanich P, Jarvis JN, Centola M (2004). Hypervariable genes – experimental error or hidden dynamics. Nucleic Acids Res.

[B21] Johnson R, Wichern D (2002). Applied Multivariate Statistics.

[B22] Peters AM, Bertram P, Gahr M, Speer CP (1993). Reduced secretion of interleukin-1 and tumor necrosis factor-alpha by neonatal monocytes. Biol Neonate.

[B23] Marwitz PA, Tenbergen-Meekes AJ, Heijnen CJ, Rijkers GT, Zegers BJ (1990). Interleukin 1 in the in vitro antigen-induced antibody response in the human adult and newborn. Scand J Immunol.

[B24] Vanden Eijnden S, Goriely S, De Wit D, Goldman M, Willems F (2006). Preferential production of the IL-12(p40)/IL-23(p19) heterodimer by dendritic cells from human newborns. Eur J Immunol.

[B25] Sullivan SE, Staba SL, Gersting JA, Hutson AD, Theriaque D, Christensen RD, Calhoun DA (2002). Circulating concentrations of chemokines in cord blood, neonates, and adults. Pediatr Res.

[B26] Juliano RL (2002). Signal transduction by cell adhesion receptors and the cytoskeleton: functions of integrins, cadherins, selectins, and immunoglobulin-superfamily members. Annu Rev Pharmacol Toxicol.

[B27] Swetman CA, Leverrier Y, Garg R, Gan CH, Ridley AJ, Katz DR, Chain BM (2002). Extension, retraction and contraction in the formation of a dendritic cell dendrite: distinct roles for Rho GTPases. Eur J Immunol.

[B28] Gillan L, Matei D, Fishman DA, Gerbin CS, Karlan BY, Chang DD (2002). Periostin secreted by epithelial ovarian carcinoma is a ligand for alpha(V)beta(3) and alpha(V)beta(5) integrins and promotes cell motility. Cancer Res.

[B29] Hamilton BJ, Burns CM, Nichols RC, Rigby WF (1997). Modulation of AUUUA response element binding by heterogeneous nuclear ribonucleoprotein A1 in human T lymphocytes. The roles of cytoplasmic location, transcription, and phosphorylation. J Biol Chem.

[B30] Morand EF, Bucala R, Leech M (2003). Macrophage inhibitory factor. Arthritis Rheum.

[B31] Roger T, Glauser MP, Calandra T (2001). Macrophage migration inhibitory factor (MIF) modulates innate immune responses induced by endotoxin and gram-negative bacteria. J Endotoxin Res.

